# The effect of ten versus twenty minutes of mindfulness meditation on state mindfulness and affect

**DOI:** 10.1038/s41598-023-46578-y

**Published:** 2023-11-24

**Authors:** Robert Palmer, Corey Roos, Nilofar Vafaie, Hedy Kober

**Affiliations:** 1 Department of Psychiatry, School of Medicine, Yale University, New Haven, CT USA; 2 Department of Psychology, Emory University, Atlanta, GA USA

**Keywords:** Psychology, Human behaviour

## Abstract

We aimed to elucidate the effects of “dose” of a single-session of mindfulness meditation on state mindfulness and affect as well as moderators of effects. 372 adults recruited remotely via Amazon’s MTurk platform were randomly assigned to either a: 10-min mindfulness meditation, 20-min mindfulness meditation, 10-min control, or 20-min control. Control conditions were recordings of a National Geographic article. Primary outcomes were changes in state mindfulness, anxiety, and negative and positive affect. Moderator variables included neuroticism, trait mindfulness, and prior meditation experience. Collapsing across doses, participants in mindfulness conditions reported greater increases in state mindfulness than in control conditions. There was a greater increase in state mindfulness in the 10-min mindfulness condition versus 10-min control condition. There were no differences between 10- and 20-min mindfulness conditions. Exploratory moderation analyses indicated that meditation (10 or 20) versus control (10 or 20) predicted increased state mindfulness among participants with lower trait mindfulness. Additionally, 20-min versus 10-min meditation predicted greater decreases in state anxiety among individuals with high trait mindfulness. Dose–response relationships were minimal, suggesting that 10 and 20 min of meditation may improve state mindfulness comparably. Findings support the benefits of brief mindfulness meditation and suggest that trait mindfulness moderates certain outcomes.

## Introduction

While mindfulness meditation was an esoteric research topic in the US just two decades ago, such research has since grown at an exponential rate^1,2^. In parallel, the percentage of American adults practicing meditation increased three-fold between 2012 and 2017, and the US meditation industry was estimated to have a revenue of $2 billion in 2020^3,4^. Reassuringly, meta-analyses of mindfulness-based interventions (MBIs) have found that these interventions can yield meaningful improvements in a wide range of measures of wellbeing^5–9^. Further, numerous studies have found that people with higher levels of trait mindfulness report lower levels of stress, depression, and anxiety^5–7^.

Yet, despite mindfulness’ entry into mainstream consciousness, many important and pragmatic questions about it remain inadequately addressed. Such questions include whether the length of a single mindfulness meditation session (e.g., 10 vs. 20 min) leads to meaningfully different outcomes, and whether individual differences moderate interventions’ effects. Such questions are important especially within a *precision medicine* approach, which aims to individualize treatments for each patient, rather than adhere to a one-size-fits-all approach. However, “prescribers” of mindfulness meditation (e.g., therapists, physicians, etc.) have limited empirical evidence to draw upon when trying to answer these practical questions.

### Prior research on dose–response relationships in mindfulness meditation and MBIs

MBIs have been shown to improve many indices of stress, cognition, and wellbeing^10,11^. In this context, it seems intuitive that “more is better,” such that people would experience greater benefits the more they practice mindfulness meditation. Indeed, a commonly-used metaphor in mindfulness teachings is that mindfulness is like a muscle that gets strengthened with practice^11^. However, research has not always supported this assumption. Several meta-analyses and a meta-regression of MBIs have reported conflicting findings regarding the moderating effects (or lack thereof) of several dose-related variables—including MBI duration, the cumulative duration of assigned or completed homework, and the number of sessions—on changes in various psychological measures^9,12–15^. Further, meta-analyses don’t allow for causal inferences regarding dose–response relationships, which is the purview of randomized controlled trials (RCTs).

To directly test the “more is better” hypothesis of the dose–response relationship, Strohmaier et al^16^ conducted a small RCT (N = 71) comparing two doses of face-to-face mindfulness practice: four 20-min versus four 5-min mindfulness meditation sessions, both administered over the course of two weeks (both were also compared to a control condition). Surprisingly, participants who engaged in the 5-min sessions reported significantly greater improvements on trait mindfulness, state mindfulness, and stress compared to participants who engaged in the 20-min sessions (with a trend in the same direction for depression and anxiety). Notably, participants in this study had limited prior experience with mindfulness practices, and other researchers have speculated that longer practices may be difficult for novice meditators^17^.

In another small RCT (N = 77), Berghoff et al^18^ randomized participants to complete two weeks of daily 10-min meditation sessions versus daily 20-min meditation sessions (the first session was in person, the rest were completed at home). The only between-group difference was greater improvement in self-compassion in the 20-min group versus the 10-min group. However, a major shortcoming of this study is that, due to low adherence, there were no significant between-group differences in the total time spent meditating, which severely limits the interpretation of results.

Taken together, these findings suggest an uncertain—and potentially counterintuitive—understanding of dose–response relationship. Further, we are unaware of any prior research that has assessed dose–response relationships for a single session of mindfulness meditation. Such an investigation may provide key insights into how to optimize the design of multi-session MBIs.

### Prior research on individual-level moderators of MBIs

Studies and meta-analyses have tried to determine which participant characteristics moderate outcomes of MBIs, however, meta-analyses suggest that findings have been inconsistent^19^. Specifically, several studies have tested trait mindfulness and the Big Five personality traits as moderators, and results have been mixed. Whereas one study (N = 131) found that lower baseline levels of acceptance and decentering predicted greater decreases in stress from MBSR (potentially because there’s more “room for improvement”), the same study found that higher levels of awareness and lower decentering predicted greater reductions in negative affect^20^. Additionally, another study of MBSR (N = 30) found that high levels of trait mindfulness predicted better outcomes (e.g., greater subjective wellbeing and lower stress and depression)^21^. Conversely, another study (N = 322) found that trait mindfulness did not moderate the effects of MBSR^22^. Further, another study (N = 288) found that neuroticism and conscientiousness—but not trait mindfulness—moderated certain effects of MBSR, whereby higher levels of neuroticism predicted larger improvements in mental distress and subjective wellbeing, while higher conscientiousness predicted less study stress^23^.

Studies of single-session MBIs have also reported mixed results. For example, one study (N = 202) reported that higher levels of trait mindfulness predicted better stress regulation following an MBI^24^, while another study (N = 76) found that low levels of the describing and observing aspects of trait mindfulness predicted greater reductions in anxiety^25^. Concerning the Big Five personality traits, two studies (N = 37, N = 56) found that neuroticism moderated attention-related outcomes, such that participants lowest in neuroticism experienced the greatest improvement in attention-related outcomes following the MBI^26^.

Prior meditation experience is another potential moderator. Multiple studies have found that prior meditation experience moderates effects of MBIs, with one study (N = 299) finding that previous meditation experience predicted greater levels of state mindfulness following a single-session MBI . Another study (N = 203) found that changes in positive affect (but not negative affect) in response to a single-session MBI were moderated by prior meditation experience, such that meditation-naïve participants reported decreases in positive affect, while meditation-experienced participants reported no such decreases in positive affect^29^. Overall, these findings leave many important questions open to further investigation.

### Current study & hypotheses

The current study aimed to help researchers and clinicians better apply the principles of precision medicine to the design of MBIs. Towards that end, we conducted a randomized controlled experiment to evaluate dose–response relationships and moderators of the effect of a single-session mindfulness meditation session. We specifically compared 10 versus 20 min of mindfulness meditation versus control conditions, and tested their effects on outcomes including state mindfulness, anxiety, negative affect, and positive affect. Participants were randomly assigned to either a: (1) 10-min mindfulness meditation, (2) 20-min mindfulness meditation, (3) 10-min control condition, or (4) 20-min control condition. Moderator variables included trait mindfulness, neuroticism, and prior meditation experience. By assessing acute changes in state mindfulness and affect, this study may help elucidate the proximal mechanisms by which MBIs with meditations of different lengths may exert their beneficial effects.

We hypothesized that both meditation conditions would yield significant improvements in state mindfulness, state anxiety, and negative affect relative to control conditions, with no effect on positive affect. We expected no difference in positive affect because prior research has shown that changes in positive affect in a single-session MBI were moderated by prior meditation experience. Given that we expected the sample to be comprised of meditation-naïve individuals as well as individuals with prior meditation experience, we expected that, at an aggregate level, we would not see significant changes in positive affect. We also hypothesized that participants in the 20-min meditation condition would report greater improvements in state mindfulness, state anxiety, and negative affect, but not positive affect, relative to participants in the 10-min meditation condition. Although this hypothesis may seem to contradict the aforementioned findings of Strohmaier et al., specifically that five minutes of meditation resulted in greater increases in state mindfulness than did twenty minutes, the sample from that study was comprised entirely of people with either no or limited prior meditation experience. We expected to recruit a more diverse sample with respect to prior meditation experience (i.e., more prior meditation experience at an aggregate level), and therefore hypothesized that there would be a dose–response relationship in favor of longer durations of meditation for all outcomes, except for positive affect. Relatedly, we hypothesized that participants’ prior meditation experience would interact with “dose,” such that people with prior meditation experience would experience greater improvements in state mindfulness, state anxiety, negative affect, and positive affect from the 20-min meditation compared to meditation-naïve individuals.

## Methods

### Participants

**Figure 1:**
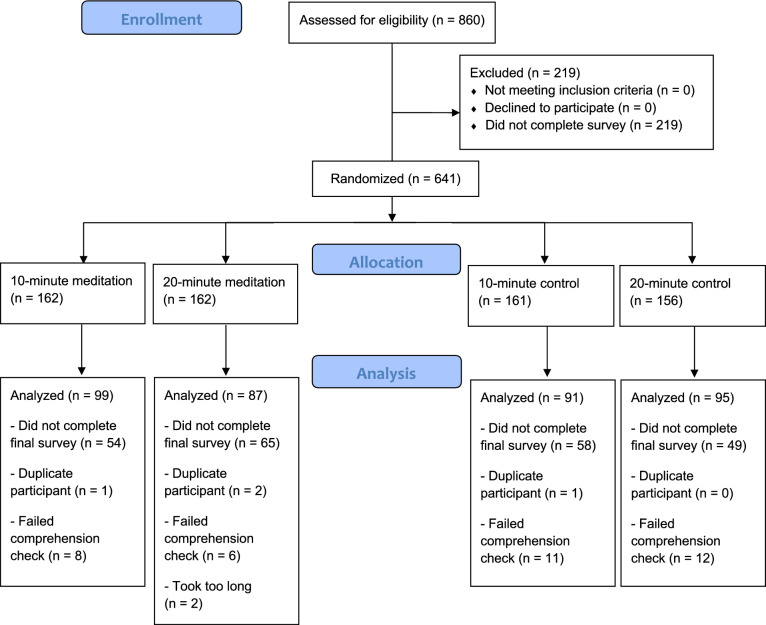
CONSORT diagram depicting participant recruitment, allocation, exclusion, and analysis.

Eight hundred and sixty participants were recruited from the United States using Amazon’s Mechanical Turk (MTurk) survey platform. Participants were excluded from the study if they were younger than 18-years old or older than 56-years old. Of the 860 participants who signed the informed consent and began the study, only 641 participants completed the first survey and were randomized (see Fig. 1 for CONSORT diagram). Of these, 162 participants were randomized to listen to the 10-min mindfulness recording, 162 participants to the 20-min mindfulness recording, 161 participants to the 10-min control recording, and 156 participants to the 20-min control recording. Of those, only 426 participants completed the study. Of these 426 participants, 54 participants were removed for failing to meet pre-determined quality control checks (e.g., “approximately how long was the recording”), which made for a final sample of 372 participants (see Table 1 for participant demographics for the entire sample and each condition).

**Table 1:** Demographic information by group.

	Whole Sample	Control10	Control20	Meditation10	Meditation20
N	372	91	95	99	87
Age M (SD)	36.5 (9.5)	37.7 (10.4)	35.1 (8.8)	35.8 (8.8)	37.5 (10.1)
Gender (% Female)	60.5	61.5	63.2	51.5	66.7
Ethnicity (%)	White 76.6	White 80.2	White 78.9	White 74.7	White 72.4
	Black 8.8	Black 11.0	Black 8.4	Black 14.1	Black 11.5
	Asian 8.3	Asian 5.5	Asian 9.5	Asian 8.1	Asian 10.3
	Other 6.3	Other 3.3	Other 3.2	Other 3.1	Other 5.8
Prior meditation experience (% Yes)	48.9	49.5	44.2	53.5	48.3
Years of education M (SD)	15.5 (2.5)	15.3 (2.6)	15.6 (2.4)	15.6 (2.5)	15.5 (2.6)
Neuroticism M (SD)	2.79 (1.00)	2.93 (1.06)	2.88 (0.96)	2.57 (1.01)	2.80 (0.95)
Trait mindfulness M (SD)	2.88 (0.51)	2.80 (0.53)	2.86 (0.51)	2.93 (0.53)	2.94 (0.47)

% indicates percent of participants, M indicates mean, SD indicates standard deviation.

### Procedures

The study was presented as an investigation of the effects of auditory attention on cognitive performance. Participants signed an online informed consent, after which each participant completed questions assessing demographic information, neuroticism, trait mindfulness, state mindfulness, positive and negative affect, and state anxiety, all using Likert scales (see below for additional details). Participants were then randomly assigned to one of the four conditions, which determined which audio recording they would listen to. Before listening to their assigned recording, participants were instructed to ensure that they were in a quiet environment with minimal distractions. To further encourage attentiveness, participants were told that they would need to answer questions based on the recording in order to be compensated for the study. Following the audio recording, which were delivered using Inquisit software, participants again answered questions assessing state mindfulness, positive and negative affect, and state anxiety, as well as multiple-choice questions and Likert scales to assess their comprehension of the recording, enjoyment of the recording, and prior meditation experience. Participants also answered open-response questions regarding their perception and understanding of the recording.

### Experimental conditions

Participants were randomly assigned to listen to one of the four aforementioned audio recordings. Versions of both the mindfulness and control recordings have been used and described previously^27^. The mindfulness meditation recordings guided participants through a typical breath-focused mindfulness meditation, instructing participants to attend to the sensations of breathing, and to practice acceptance of the moment and of any distraction without judgment (modeled after typical mindfulness instructions delivered in MBSR). The control condition was a recording of a National Geographic article about the history of sequoia trees and Sequoia National Forest. Notably, both the mindfulness and control conditions were recorded by the same person, had similar rates of speech and word frequencies, included the same number of words, and had periods of silence at the same times and for the same duration. Additionally, all recordings began by instructing the listener to adopt a comfortable and upright posture. The only difference between the 10- and 20-min recordings was the addition of periods of silence at various points during the 20-min versions. Participants took an average of 30 min to complete the study and were paid $2 as compensation; participants in the 20 versus 10 min conditions (both control and mindfulness) took, on average, 10 min longer to complete the study. This study was approved by Yale University Institutional Review Board.

### Measures

Immediately before the recording, participants completed measures of demographics, neuroticism, trait mindfulness, positive and negative affect, state mindfulness, and state anxiety (see below). Immediately after the recording, participants completed the same measures of positive and negative affect, state mindfulness, and state anxiety as well as measures assessing their perception of the recording, their attention to and comprehension of the recording, and prior meditation experience (see below).

#### Demographic survey

Before the intervention, participants were asked to report their date of birth, gender, race, ethnicity, educational attainment, and marital status.

#### Neuroticism

Neuroticism was assessed before the intervention using the neuroticism subscale of the Big Five Personality Inventory^30^. The neuroticism subscale consists of eight items (α = 0.90) and uses a five-point Likert scale (1 = *disagree strongly*, 5 = *agree strongly*). Each item began with the phrase “I see myself as someone who…” Sample items for the neuroticism subscale include: “worries a lot” and “is emotionally stable, not easily upset,” with the latter item reverse-coded.

#### Trait mindfulness

Trait mindfulness was assessed using the Cognitive and Affective Mindfulness Scale-Revised (CAMS-R)^31^. The CAMS-R is a 12-item self-report measure (α = 0.66) that uses a four-point Likert scale (1 = *rarely/not at all*, 4 = *almost always*). Sample items include: “It’s easy for me to keep track of my thoughts and feelings” and “I try to notice my thoughts without judging them.”

#### Positive and negative affect

Positive and negative affect were measured using the International Positive and Negative Affect Schedule Short Form (I-PANAS-SF)^32^. The I-PANAS-SF is a self-report measure comprised of 10 items, five of which refer to positive affect and five of which refer to negative affect. It uses a five-point Likert scale (1 = *not at all*, 5 = *a great deal*). Participants were instructed to rate the extent to which they felt each emotion in the present moment. Sample items for positive affect include: “Attentive” and “Active.” Sample items for negative affect include: “Ashamed” and “Nervous.” For positive affect, Cronbach’s alphas were 0.83 and 0.87, pre- and post-intervention, respectively. For negative affect, Cronbach’s alphas were 0.82 and 0.89, pre- and post-intervention, respectively.

#### State mindfulness

State mindfulness was measured using the State Mindfulness Scale (SMS)^33^. The SMS is a self-report measure comprised of 21 items and uses a five-point Likert scale (1 = *not at all*, 5 = *very well*). The measure assesses state mindfulness of body (e.g., “I noticed physical sensations come and go”; six items) and mind (e.g., “I was aware of what was going on in my mind”; fifteen items), though the subscales were summed in the present study. Participants were instructed to, “Please use the rating scale to indicate how well each statement describes your experiences in the past 10 min.” Cronbach’s alphas were 0.95 and 0.96, pre- and post-intervention, respectively.

#### State anxiety

State anxiety was measured using the five-item State scale of the Spielberger State-Trait Anxiety Inventory (STAI-S-5)^34^. The STAI-S-5 uses a four-point Likert scale (1 = *not at all*, 4 = *very much so*). Participants were instructed to rate how well each item described how they felt in the present moment. Sample items included: “I feel nervous” and “I feel upset.” Cronbach’s alphas were 0.88 and 0.81, pre- and post-intervention, respectively.

#### Perception of the intervention

To assess participants’ perception of the interventions and their overall acceptability, participants answered two 5-point Likert scales assessing the extent to which they enjoyed the recording and to what extent they found listening to the recording to be a worthwhile experience.

#### Attention checks

To assess participants’ attentiveness and comprehension, participants were required to correctly provide a numerical code given at the end of the recording. Participants were then asked multiple-choice questions about the language of the speaker and the approximate duration of the recording. Additionally, participants were asked to write complete sentence answers to the following two open-ended questions: 1) “What was the recording about?” and 2) “What did you think of the recording?”.

#### Prior meditation experience

To assess participants’ prior experience with mindfulness meditation, participants were asked whether or not they had previously tried mindfulness meditation. If they answered that they did, we followed this with five additional questions to assess the extent of their experience.

### Statistical analysis

The data were initially assessed to remove duplicate participants as well as participants who took over two hours to complete the study and those who incorrectly answered questions assessing attentiveness and comprehension. Data were analyzed using SPSS version 28. To assess within- and between-condition differences, we performed repeated-measures ANOVAs on state mindfulness, state anxiety, negative affect, and positive affect. Time (i.e., pre- vs. post-intervention) was entered as a within-subjects factor, while Condition (i.e., meditation vs. control) and Duration (i.e., 10 vs. 20 min, or “dose”) were entered as between-subjects factors. Individual within- and between-group differences were subsequently assessed with paired or independent-samples t-tests (note that we did not directly compare unmatched pairs of conditons, e.g., 20-min control vs. 10-min mindfulness).

To evaluate moderators, we conducted moderated regression analyses using the PROCESS 4.0 macro (Model 1) with 5,000 bootstrapped samples^35^. Moderator variables included trait mindfulness, neuroticism, and prior meditation experience. For the moderated regression analyses, we were primarily interested in moderators of the effect of mindfulness meditation (10- or 20-min) versus control (10- or 20-min) on outcomes, as well as the effect of the effect of meditation duration (20- vs. 10-min mindfulness meditation only) on outcomes. Hence, we created two dummy-coded variable to represent these specific comparisons. We first conducted a series of moderated regression models with mindfulness meditation (10- or 20-min) versus control (10- or 20-min) as the predictor variable. Subsequently, we conducted a series of regression models with 20- versus 10-min mindfulness meditation as the predictor variable.

To probe the nature of statistically significant moderation effects, we applied the Johnson-Neyman (JN) technique^36^ in the PROCESS 4.0 macro in SPSS, as an exploratory analysis. The JN technique does not require the analyst to select specific values of the moderator variable to test varying effect of the predictor variable on the outcome. Rather, the JN techniques computes ‘transition points’ across continuum of values for the moderator variable in which the effect of the predictor variable transitions between statistically significant and nonsignificant. The JN technique can be applied to determine a “region of significance” or the specific range of values of the moderator variable in which the effect of the predictor variable on the outcome is statistically significant.

### Ethical approval

Approval was obtained from the ethics committee of Yale University. The procedures used in this study adhere to the tenets of the Declaration of Helsinki.

### Consent to participate

Informed consent was obtained from all individual participants included in the study.

## Results

### Participant characteristics

Table 1 shows participant characteristics by group. We conducted ANOVAs and Chi Square tests and found no significant differences between groups on any of the baseline variables (all *ps* > 0.06).

### Interventions effects: state mindfulness

**Figure 2:**
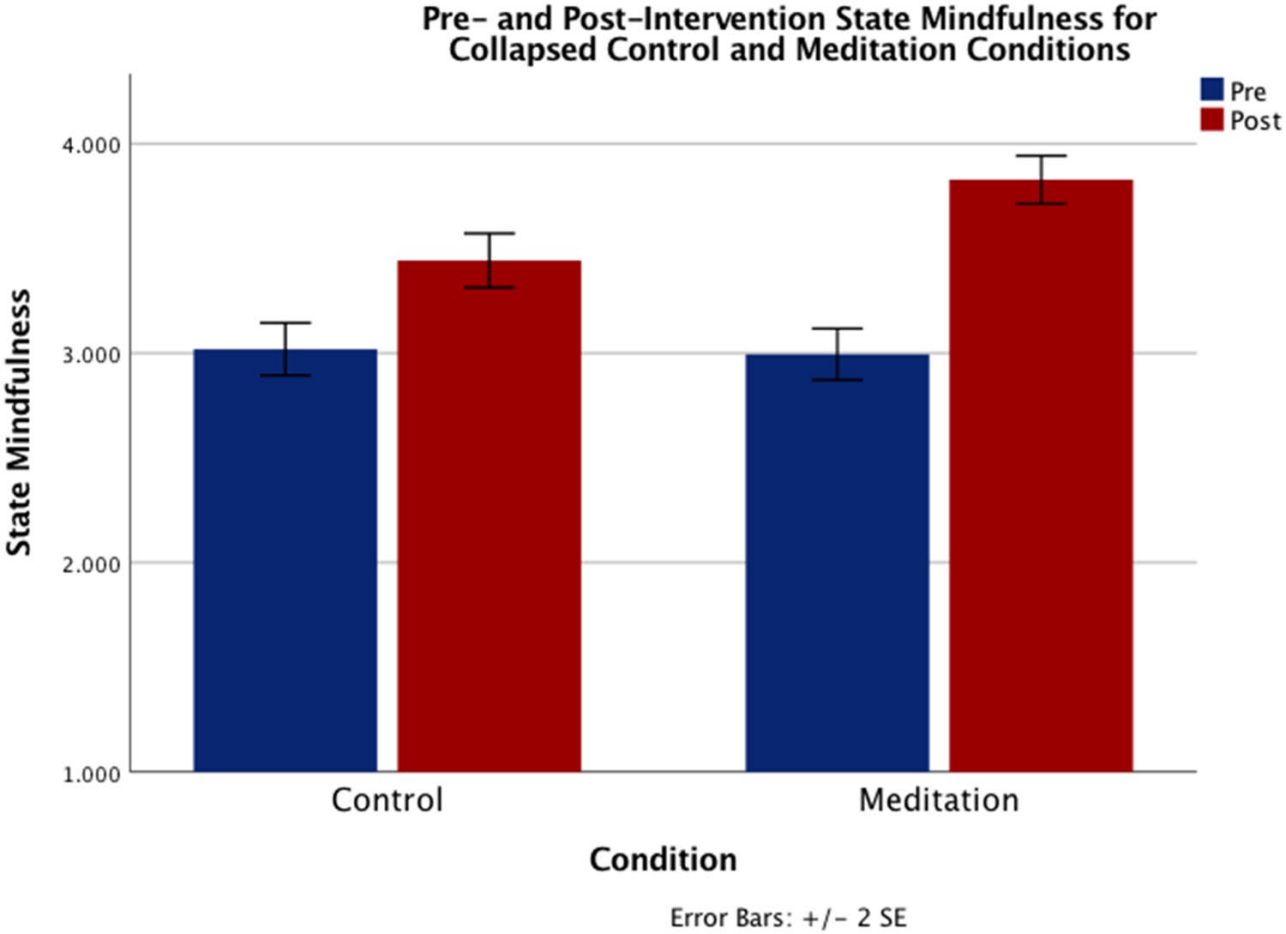
Mean pre- and post-intervention values of state mindfulness for the collapsed control and meditation conditions. Error bars represent standard errors.

There was a significant main effect of Time (*F*_(1,368)_ = 102.99, *p* < 0.001, *η*^2^*p* = 0.219), such that participants across all conditions reported an increase in state mindfulness post- compared to pre-intervention (see Table 2 for means and standard deviations for each condition as well as within- and between-group comparisons). Importantly, this main effect was qualified by a significant Time x Condition interaction (*F*_(1,368)_ = 10.93, *p* < 0.005, *η*^2^*p* = 0.029). Specifically, participants in the mindfulness conditions reported a significantly greater increase in state mindfulness than did participants in the control conditions (*t*_(370)_ = 3.33, *p* < 0.001; See Fig. 2). Notably, post-hoc tests showed the change in state mindfulness in the 10-min meditation condition was greater than that of the 10-min control condition (*t*_(188)_ = -3.18, *p* < 0.005). There were no other significant main effects or interactions, including none of the predicted interactions with Duration (all *ps* > 0.27).

**Table 2:** Means and standard deviations (in parentheses) for pre- and post-intervention measures for all conditions as well as Cohen’s d for within-group (pre vs. post) differences.

	Control10 (n = 91)			Control 20 (n = 95)			Meditation 10 (n = 99)			Meditation 20 (n = 87)		
	Pre	Post	Cohen’s d	Pre	Post	Cohen’s d	Pre	Post	Cohen’s d	Pre	Post	Cohen’s d
State mindfulness	3.06 (.86)	3.41** (.85)	0.32	2.98 (.85)	3.47*** (.91)	0.41	2.92 (.81)	3.81*** (.80)	**0.71**	3.07 (.86)	3.85*** (.76)	0.65
State anxiety	1.32 (.43)	1.17* (.42)	− 0.25	1.28 (.51)	1.19 (.35)	− 0.15	1.27 (.54)	1.12* (.34)	− 0.23	1.21 (.35)	1.09** (.23)	− 0.29
Negative affect	1.38 (.52)	1.20* (.63)	− 0.22	1.32 (.60)	1.29 (.63)	− 0.04	1.29 (.57)	1.17 (.52)	− 0.16	1.29 (.54)	1.10** (.27)	− 0.31
Positive affect	3.25 (.81)	3.10 (.96)	− 0.12	3.26 (.96)	3.04 (1.03)	− 0.16	3.31 (.90)	3.24 (1.03)	− 0.05	3.43 (.90)	3.30 (.96)	− 0.10

*Indicates a within-group difference at *p* < 0.05, **p < 0.01, and ***p < 0.001. Bold numerals indicate a statistically significant difference between a meditation condition and its control condition counterpart (*p* < 0.001).

### Interventions effects: state anxiety

There was a significant main effect of Time (*F*_(1,368)_ = 17.94, *p* < 0.001, *η*^2^*p* = 0.046), such that participants across conditions exhibited decreases in state anxiety. There were no other significant main effects or interactions, including none of the predicted interactions with Duration (all *ps* > 0.54). The change in state anxiety was not significantly different between any conditions (all *ps* > 0.56; see Table 2).

### Interventions effects: negative affect

There was a significant main effect of Time (*F*_(1,368)_ = 10.97, *p* = 0.001, *η*^2^*p* = 0.029), such that participants across conditions exhibited a decrease in negative affect. There were no other significant main effects or interactions (all *ps* > 0.16). The change in negative affect was not significantly different between any conditions (all *ps* > 0.13; see Table 2).

### Interventions effects: positive affect

There was a significant effect of Time (*F*_(1,368)_ = 4.33, *p* = 0.04, *η*^2^*p* = 0.012), such that participants across all conditions exhibited a decrease in positive affect. There were no additional significant main effects or interactions (all *ps* > 0.51; see Table 2).

### Moderated regression analyses

**Figure 3:**
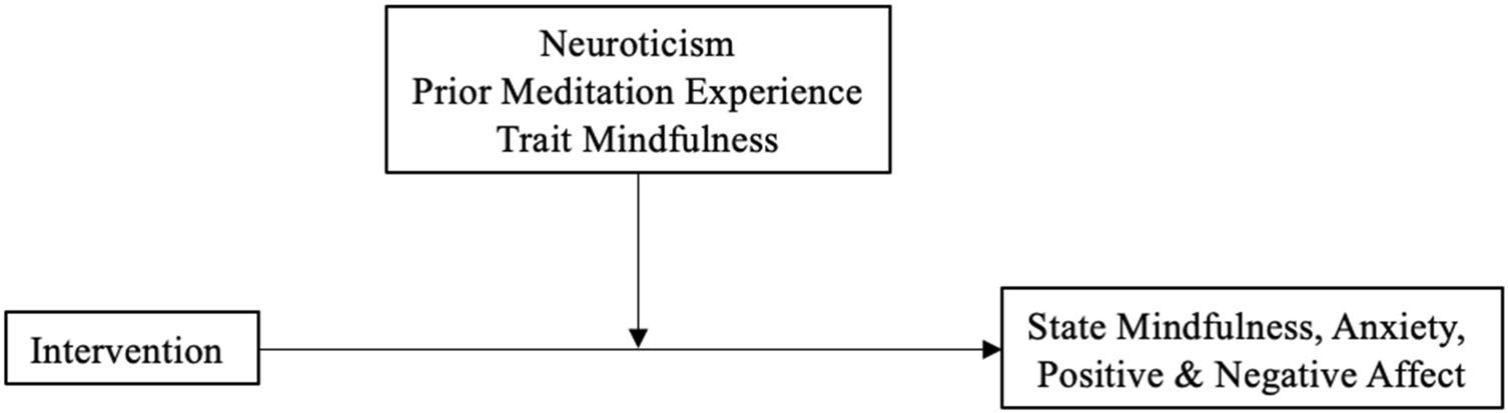
Conceptual diagram of the moderation analyses. Intervention was the independent variable, neuroticism, prior meditation experience, and trait mindfulness were moderating variables, and state mindfulness, anxiety, and positive and negative affect were dependent variables.

**Figure 4:**
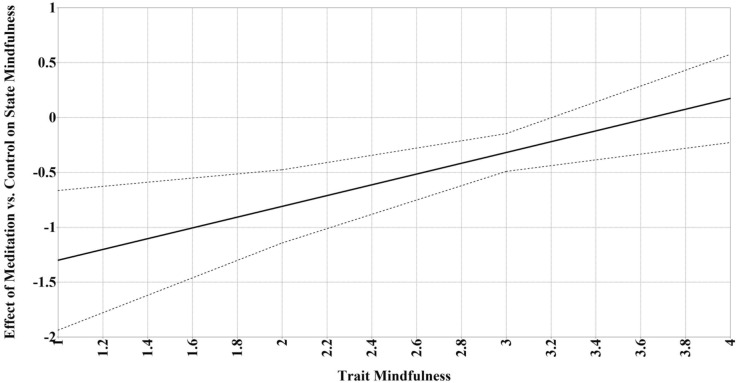
Trait mindfulness moderated the effects of meditation (mindfulness conditions vs. control) on changes in state mindfulness. Dotted lines represent 95% Johnson-Neyman confidence intervals. Region of significance is where the confidence intervals do not cross zero and is below 3.23, such that mindfulness meditation versus control predicted significantly greater increases in state mindfulness when trait mindfulness was below 3.23.

**Figure 5:**
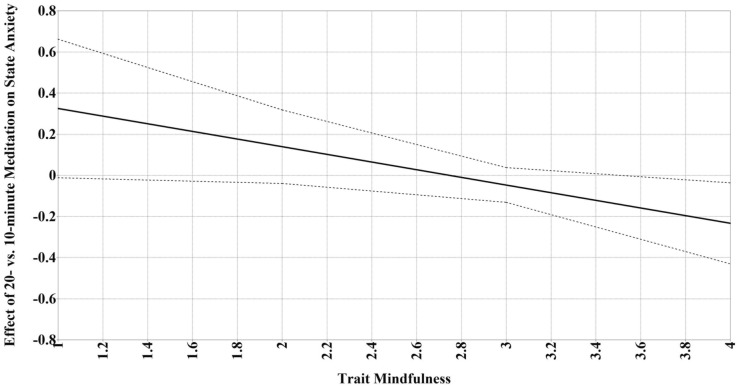
Trait mindfulness moderated the effects of meditation duration (20- vs. 10-min mindfulness meditation) on changes in state anxiety. Dotted lines represent 95% Johnson-Neyman confidence intervals. Region of significance is where the confidence intervals do not cross zero and was above 3.48, such that the 20- versus 10-min mindfulness meditation predicted greater decreases in state anxiety when trait mindfulness was above 3.48.

Figure 3 presents a conceptual diagram of the moderation analyses. Trait mindfulness moderated the effect of meditation (10- or 20-min) versus control (10- or 20-min) on changes in state mindfulness (*p* = 0.004), such that meditation led to greater increases in state mindfulness only among participants with lower trait mindfulness (below 3.23; *d* = 0.50; *p* < 0.05; 73.1% of sample; Fig. 4). Additionally, trait mindfulness moderated the meditation duration effect (20-min vs. 10-min meditation) on changes in state anxiety (*p* = 0.03), such that 20 min of meditation led to greater decreases in state anxiety only among participants with higher trait mindfulness (above 3.48; *d* = 0.35; *p* < 0.05; 23.1% of sample; Fig. 5). There were no other significant moderation effects (i.e., no effects of meditation experience or neuroticism; see Tables 3 and 4).

**Table 3:** Results of moderated regression analyses with meditation (10- or 20-moinute) versus control (10- or 20-min) as the predictor variable.

		State mindfulness			State anxiety			Negative affect			Positive affect		
		Coefficient	SE	*p*	Coefficient	SE	*p*	Coefficient	SE	p	Coefficient	SE	*p*
Meditation versus control	Neuroticism	− 0.17	0.09	0.06	0.02	0.04	0.5	0.06	0.06	0.3	0.05	0.1	0.61
	Trait mindfulness	**0.49**	**0.17**	**0.004**	0.01	0.07	0.88	− 0.1	0.11	0.34	0.11	0.2	0.59
	Meditation experience	− 0.23	0.17	0.19	− 0.01	0.07	0.9	0.13	0.11	0.22	− 0.09	0.21	0.68

Bolded numerals indicate a statistically significant interaction.

**Table 4:** Results of moderated regression analyses with 20- versus 10-min mindfulness meditation as the predictor variable.

		State mindfulness			State anxiety			Negative affect			Positive affect		
		Coefficient	SE	*p*	Coefficient	SE	*p*	Coefficient	SE	p	Coefficient	SE	*p*
Meditation10 versus Meditation20	Neuroticism	0.11	0.12	0.36	0.07	0.05	0.1	0.1	0.06	0.11	0.11	0.12	0.36
	Trait mindfulness	− 0.09	0.23	0.7	− **0.19**	**0.09**	**0.03**	− 0.23	0.12	0.06	− 0.09	0.23	0.7
	Meditation experience	− 0.24	0.23	0.31	− 0.02	0.09	0.79	0.08	0.12	0.51	− 0.24	0.23	0.31

Bolded numerals indicate a statistically significant interaction.

### Perception of interventions

Participants completed two Likert scales to assess how much they enjoyed listening to the recording and how worthwhile they found the experience. Responses were averaged to create an estimate of the participant’s overall positive perception of the recording. Mean positive perception did not differ between the two 10-min conditions (*t*_(188)_ = − 1.26, *p* = 0.22), nor between the two meditation conditions (*t*_(184)_ = 0.15, *p* = 0.88). There were significant differences between the two control conditions, with the 10-min control recording being rated more favorably (*t*_(184)_ = 2.46, *p* = 0.02), as well as between the two 20-min conditions, with the meditation recording being rated more favorably (*t*_(180)_ = − 3.34, *p* = 0.001).

## Discussion

Consistent with our hypotheses, mindfulness meditation led to a significantly greater increase in state mindfulness compared to the control conditions. Further, additional analyses revealed that this effect was moderated by trait mindfulness, such that meditation (relative to control) increased state mindfulness especially among participants with lower trait mindfulness. This finding suggests that individuals with relatively low trait mindfulness have more “room for improvement” with regards to state mindfulness, perhaps akin to how people beginning an exercise program for the first time can experience relatively fast changes in physical fitness with even modest effort^37^.

Notably, contrary to our hypotheses, we found no significant differences between meditation and control conditions on anxiety or negative affect. We were also surprised to find no differences between the 10- and 20-min meditation conditions, suggesting that a higher “dose” of meditation was not associated with overall greater changes in any of the outcomes measured in this study. However, exploratory moderation analyses found that meditating for 20 versus 10 min led to greater decreases in state anxiety for participants with higher trait mindfulness. Taken together, these findings suggest that 10-min of mindfulness meditation may be just as effective as 20-min in improving state mindfulness for the majority of people, which runs counter to the popular notion that “more is always better.” Indeed, given the often-used metaphor of mindfulness being a muscle that’s strengthened with practice, these findings may come as a surprise. Nevertheless, it is possible that typical-dose response relationships may exist at higher doses than measured in this study, and/or that 10 and 20 min of meditation are close to each other on the dose–response curve (at least for this sample). It is also possible that the dose–response relationship for MBIs follow an inverted U-shaped curve^38^. Thus, future research could test additional durations to better understand the nature of dose–response relationships in single sessions of meditation.

Another interpretation is suggested by the finding of Strohmaier et al^17^, who previously found that four 5-min meditation sessions over two weeks—as compared to four 20-min sessions over two weeks—resulted in greater increases in state and trait mindfulness and greater decreases in stress, with similar trends for depression and anxiety. Taken together with the current findings, Strohmaier’s findings may suggest that some dose–response relationships with MBIs require multiple meditation sessions to manifest to a statistically significant degree. This hypothesis is in line with the popular notion that MBIs cultivate new habits and qualities of mind that must be practiced repeatedly in order to be deeply learned and embodied, much like learning to play an instrument. As such, it may be that single-session MBIs induce a limited degree of consolidation of these new skills and that these changes are strengthened with repeated practice over multiple sessions.

Another important consideration is that the lack of differences between meditation and control may have been due in part to a floor effect, since 55% and 56.5% of participants reported the lowest possible level of state anxiety and negative affect, respectively, pre-intervention. Indeed, many studies of single-session mindfulness meditations have induced anxiety or rumination in participants before or after instructing them to practice mindfulness, thereby increasing the “room for improvement”^39–41^. Relatedly, it’s conceivable that dose–response relationships may have appeared had these sorts of provocations been incorporated.

The findings of the current study also suggest that the optimal duration of a single meditation session may vary according to an individual’s level of trait mindfulness. Moderation analyses indicated that meditating for 20 min (vs. 10 min) predicted greater decreases in state anxiety among individuals with relatively higher trait mindfulness. There could be several possible interpretations for this finding, including the possibility that individuals higher in trait mindfulness may benefit more from longer periods of practice relative to those low in trait mindfulness. Expressed another way, it may be beneficial to titrate the duration of a given session of meditation according to an individual’s level of trait mindfulness, in the same way that one might titrate the duration of a workout to an individual’s level of physical fitness. That said, moderation analyses did not reveal a similar trend for other outcomes, raising the possibility that this finding may be due to error. Additionally, it is notable that prior research that used a different measure for trait mindfulness found that high levels of certain facets of mindfulness and low levels of other facets were associated with better improvements in state anxiety in a single-session MBI^28^. However, given the use of different measures of trait mindfulness, it is difficult to compare these findings and draw conclusions. Thus, further research with multiple mindfulness measures and larger samples is warranted to replicate the current findings and to disambiguate these effects.

Contrary to our expectations, prior meditation experience did not moderate the effects of the interventions. This finding may be at odds with the results of a prior study that found that participants with prior meditation experience had higher levels of state mindfulness following an eight-minute, single-session MBI as compared to meditation-naïve participants^28^. One possible explanation for these differing findings is that the participants with prior meditation experience in the aforementioned study could have had a different amount of prior meditation experience than did participants in the current study. This possibility highlights the importance of developing a standardized way for assessing participants’ prior meditation experience, thereby enabling more precise and effective comparisons within and between studies. Nevertheless, our finding suggests that meditation-naïve participants can follow mindfulness instructions and experience meaningful improvements from a single meditation to a degree comparable to people with prior experience. Additionally, it seems that both 10 and 20 of minutes of meditation are reasonable durations of meditation for meditation-naïve individuals.

It is also noteworthy that neuroticism did not moderate the interventions’ effects. Prior research using the same ten-minute meditation and control scripts found that neuroticism moderated the interventions’ effects on measures of attention^27^, such that participants with lower neuroticism tended to perform better on various measures of attention following a ten-minute meditation than did participants with higher neuroticism. Additionally, other research has found that neuroticism moderated the effects of a four-session meditation training, such that participants with higher neuroticism experienced greater reductions in negative mood, perceived stress, state anxiety, and psychological distress^42^. It may be the case that neuroticism moderates the effects of single-session interventions only for certain outcomes (e.g., attention), whereas neuroticism’s moderation of multi-session interventions’ effects may include additional outcomes as well (e.g., mood and anxiety). Consequently, further research on the moderating effects of neuroticism on single- and multi-session mindfulness interventions is warranted.

Since the only significant difference between the meditation and control conditions in the current study was a greater increase in state mindfulness in the meditation conditions, it is possible that acute changes in state mindfulness, and not acute changes in affect, could be a proximal mechanism of action by which MBIs exert their beneficial effects on mental wellbeing, as other have previously suggested^43–45^. Indeed, while one study on MBIs did not support the mediating role of state mindfulness^17^, other studies have found that state mindfulness mediates a variety of positive outcomes of both single- and multi-session MBIs, including positive emotions, hope, gratitude, trait mindfulness, and psychological distress^46–48^.

Given the mainstream perception of mindfulness as a stress-relieving panacea, the large degree to which the effects of the control conditions resemble those of the meditation conditions in this study may be surprising. The control conditions’ anxiolytic and mindfulness-boosting effects may have been due to a combination of factors, including the speaker’s soothing voice, the potentially anxiolytic content of the audio recording (i.e., the history of sequoia trees), and the intermittent periods of silence. Further, these findings are fairly consistent with prior research comparing mindfulness meditation to sham meditation^49,50^, which supports the importance of using strong controls in mindfulness research and of identifying both specific and unique effects and mechanisms of action.

This study has multiple strengths, one of which is the diversity of the participants with respect to age, gender, and ethnicity. Many studies of MBIs have relied upon local university students or adults, which can limit the generalizability of the findings. Relatedly, the study’s sample size is larger than that of many single-session MBIs in the published literature, which further increases the generalizability of the current findings and increases the study’s power.

Another strength of this study is the use of active control conditions, specifically one that has been used in prior published research. A common criticism of mindfulness research has been the frequent use of passive control conditions, which has likely led to an inflated estimate of the effectiveness of MBIs^51–54^. Additionally, so as to help isolate the unique effects of the mindfulness components of the intervention, the control conditions in the current study were designed to match the mindfulness recordings in terms of word count, pace of speech, tone of voice, and periods of silence.

This study has several limitations. Perhaps most importantly, this study only sheds light on the effects of the duration of a single session of mindfulness meditation and may not generalize to multi-session MBIs. Further, as the outcomes were measured immediately before and after the intervention, further research would be needed to assess the longevity of any intervention’s effects. For example, although 10- and 20-min meditations may lead to comparable increases in state mindfulness immediately after the meditation, it may be that the increases persist longer in participants who completed the 20-min meditation (or vice versa).

Another limitation is that the setting in which participants completed the study was not standardized or directly supervised, as would be the case if the study were conducted in person. Indeed, although we used multiple methods to ensure participants’ attentiveness and comprehension (and excluded participants who endorsed or demonstrated poor adherence to or understanding of instructions), it is still possible that some participants who were included in the final analysis were minimally attentive. Thus, a possible explanation for the lack of a dose–response relationship is that the overall quality of meditation practice (i.e., the total time spent “actively meditating”) may not have differed between meditation conditions due to high rates of mind-wandering that may have occurred during the prolonged periods of silence in the 20-min condition. This phenomenon would be unsurprising, given that a high frequency of mind-wandering has been found in the general population^55,56^. Consequently, future studies could attempt to measure the frequency or duration of participants’ mind-wandering and on-task mindful awareness as well as meditation practice quality^57,58^.

Consistent with our hypotheses, mindfulness meditation led to a greater increase in state mindfulness compared to control conditions, an effect which was moderated by trait mindfulness. Moderation outcomes also revealed that trait mindfulness moderated the effect of meditation duration on changes in state anxiety. To our surprise, however, we found no other differences between the two durations of meditation, which raises important questions about the nature of dose–response relationships in MBIs. Additionally, neuroticism and prior meditation experience were not found to moderate effects of the interventions. The current findings provide a step towards understanding of dose–response relationships, and we hope that future research will further investigate such relationships and moderators of single- as well as multi-session MBIs.

## Data Availability

The datasets generated and analyzed during the current study are available in the Open Science Framework. https://osf.io/sw9bj/?view_only=37fd712d0c494f249c59d5ad51d962f5.
